# Plant Leaf Chlorophyll Content Retrieval Based on a Field Imaging Spectroscopy System

**DOI:** 10.3390/s141019910

**Published:** 2014-10-23

**Authors:** Bo Liu, Yue-Min Yue, Ru Li, Wen-Jing Shen, Ke-Lin Wang

**Affiliations:** 1 Nanjing Institute of Environmental Sciences, Ministry of Environmental Protection, Nanjing 210042, China; E-Mails: boxueyu_liu@hotmail.com (B.L.); swjb1982@163.com (W.-J.S.); 2 Institute of Subtropical Agriculture, Chinese Academy of Sciences, Changsha 410125, China; E-Mail: kelin@isa.ac.cn; 3 Huanjiang Observation and Research Station for Karst Ecosystems, Chinese Academy of Sciences, Hechi 547100, China; 4 Institute of Remote Sensing and Digital Earth, Chinese Academy of Sciences, Beijing 100094, China; E-Mail: liru@radi.ac.cn

**Keywords:** field imaging spectroscopy system, spectral sensor, chlorophyll, spectral analysis

## Abstract

A field imaging spectrometer system (FISS; 380–870 nm and 344 bands) was designed for agriculture applications. In this study, FISS was used to gather spectral information from soybean leaves. The chlorophyll content was retrieved using a multiple linear regression (MLR), partial least squares (PLS) regression and support vector machine (SVM) regression. Our objective was to verify the performance of FISS in a quantitative spectral analysis through the estimation of chlorophyll content and to determine a proper quantitative spectral analysis method for processing FISS data. The results revealed that the derivative reflectance was a more sensitive indicator of chlorophyll content and could extract content information more efficiently than the spectral reflectance, which is more significant for FISS data compared to ASD (analytical spectral devices) data, reducing the corresponding RMSE (root mean squared error) by 3.3%–35.6%. Compared with the spectral features, the regression methods had smaller effects on the retrieval accuracy. A multivariate linear model could be the ideal model to retrieve chlorophyll information with a small number of significant wavelengths used. The smallest RMSE of the chlorophyll content retrieved using FISS data was 0.201 mg/g, a relative reduction of more than 30% compared with the RMSE based on a non-imaging ASD spectrometer, which represents a high estimation accuracy compared with the mean chlorophyll content of the sampled leaves (4.05 mg/g). Our study indicates that FISS could obtain both spectral and spatial detailed information of high quality. Its image-spectrum-in-one merit promotes the good performance of FISS in quantitative spectral analyses, and it can potentially be widely used in the agricultural sector.

## 1. Introduction

Plants cover more than 70% of the global land surface and are among the most important resources on the Earth; their distributions are also intensively and closely related to human activities. Terrestrial plants that can perform photosynthesis are the energy and organic matter providers for almost all ecosystems and are also the main products of the vast majority of terrestrial ecosystems. The importance of plants has made the extraction of information on plants based on various means and methods a topic of constant interest [1,2]. Spectroscopic technology provides fast, convenient and non-destructive detection, so it has long been used in fields, such as crop yield assessments [3], vegetation ecology assessments [4–6], light and effective radiation [7], land productivity [8], wetland management [9] and tree species identification [10,11]. Imaging spectroscopy can be divided into two categories according to the carrying platform of the sensors and applicable fields: spectroscopy that is based on remote sensing platforms, such as satellites and aircrafts, utilizes aerospace remote sensing and is suitable for large-scale regional studies and applications; and spectroscopy that is based on small ground application platforms, such as ground-based remote sensing systems, has a compact size and is flexible, mobile and widely used in agriculture applications, such as the retrieval of plant chlorophyll content and other biochemical parameters, discrimination of crops/weeds, monitoring of crop pests and disease and quality control of agricultural products and meat [12–18].

Ground remote sensing systems can be divided into single-point sensor spectrometers and imaging spectrometers depending on whether an image can be formed. Compared with single-point sensor spectrometers, an imaging spectroscopy system can provide tens to several hundreds of spectral channels and a wealth of images and spatial details, and it can perform image analyses and spectral analyses simultaneously. Such systems can also acquire qualitative and quantitative information, as well as information on positioning, distribution and morphology. The ability to obtain “pure” pixel information not only makes up for the drawbacks of conventional non-imaging spectroscopy instruments, but also greatly expands the potential applications of imaging spectroscopy [13–18]. Ground remote sensing systems have been widely used in agriculture [12–16], food security [17], quality control [18], chemical imaging analysis [19,20], biomedicine [21] geology [22,23], color science, materials science, machine vision and other fields [24,25]. These systems have become a popular topic in the research on imaging spectroscopy technology and its applications.

Chlorophyll is one of the most important biochemical parameters of plants and is usually an indicator of plants' nutritional stress, photosynthetic capacity and the health status of plants; therefore, it is an important information parameter in research on crop quality monitoring, ecosystem productivity estimation, carbon cycles, *etc.* Fast and non-destructive chlorophyll content estimation is an important field of application for spectroscopy technologies. However, most current ground-based studies on chlorophyll retrieval have used non-imaging spectral data obtained by single-point sensors or images formed by a small number of wavelengths that are generated by multi-spectrum systems. The use of imaging spectroscopy systems, which are emerging high-tech detection systems, for the non-destructive acquisition of plant chlorophyll information must still be investigated [26–30].

The Chinese Scientific Community has exhibited strong interest in the application of small field imaging spectroscopy systems (FISSs). A series of FISSs have been developed by the Chinese Academy of Sciences, including two visible light systems and one shortwave infrared system [14,31,32]. In the present study, one of these visible FISSs (the first FISS in China that is suitable for agricultural research) was used to obtain 101 FISS images of soybean leaves and to extract their reflectance, derivative spectra and other information. The chlorophyll content was retrieved using multiple linear regression (MLR), partial least squares (PLS) regression and support vector machine (SVM) regression. In order to compare the performance of imaging spectrometer and traditional single-point sensor spectrometer, we also used an ASD spectrometer to obtain the spectral data of these soybean leaf samples simultaneously. The main purposes of the present study were to: (1) verify the performance of FISS in a quantitative analysis through the estimation of chlorophyll content and comparison with a traditional non-imaging spectrometer; (2) evaluate and compare the potential of FISS data-derived spectral reflectance and derivative spectra in estimating the chlorophyll content of plant leaves; and (3) evaluate the performance and accuracy of different estimation methods (linear and nonlinear) and determine the quantitative spectral analysis method most suitable for FISS.

## 2. FISS and Experimental Design

### 2.1. FISS

**Figure 1.**
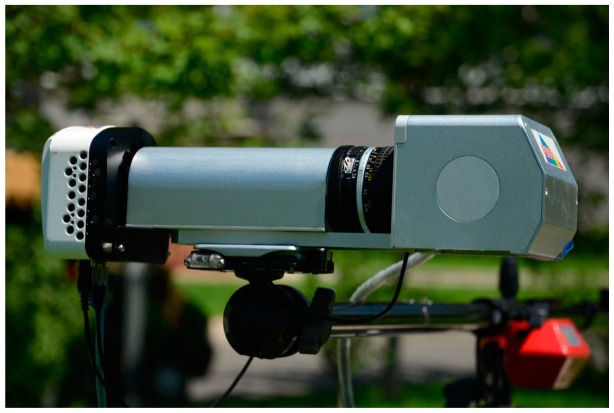
Photograph of the field imaging spectroscopy system (FISS) components.

**Figure 2.**
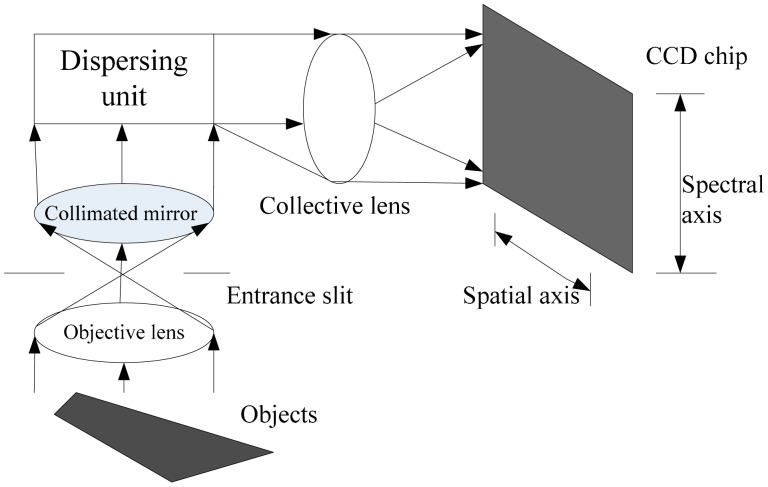
Basic principle of the FISS.

FISS consist of a multi-purpose platform, electronic system, opto-mechanical system, computer system and auxiliary equipment (Figure 1) [31]. The opto-mechanical system, which is the key component of the FISS, consists of a scanning mirror, optical lenses, spectroscopic devices (ImSpector V9, Spectral Imaging, Ltd., Qulu, Finland) and a charge-coupled device (CCD) camera. The electronic system includes the power and motor control circuits. The motor control circuit is primarily used to control the rotation of the scanning mirror, synchronize the beam splitter and receiver and collect and store the data. The basic principle of FISS is shown in Figure 2 [31].

The computer system includes hardware and software: the hardware is a portable laptop computer, and the software includes the FISS operating software, data acquisition software and data processing software. The instrument operating software and data acquisition software are used for setting the instrument parameters (integration time, aperture, field of view, cooling temperature, *etc.*) and can display images and spectra in real time. The primary functions of the data processing software are data format conversion, geometric correction, radiometric correction, image segmentation, interception and image stitching.

**Figure 3.**
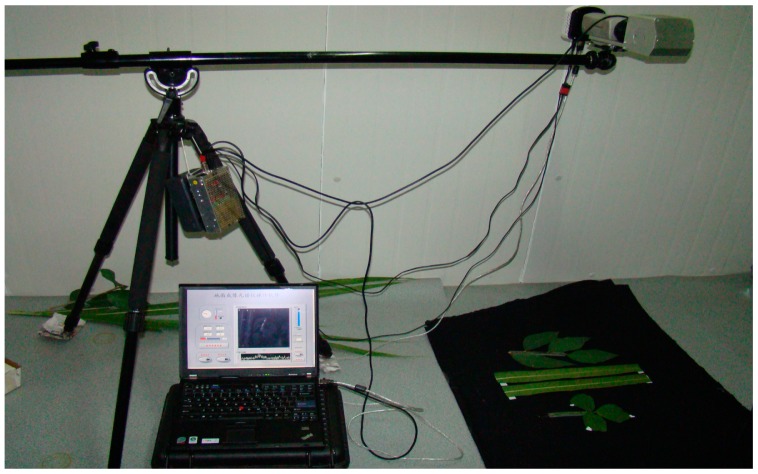
Actual photograph of FISS field measurements.

**Figure 4.**
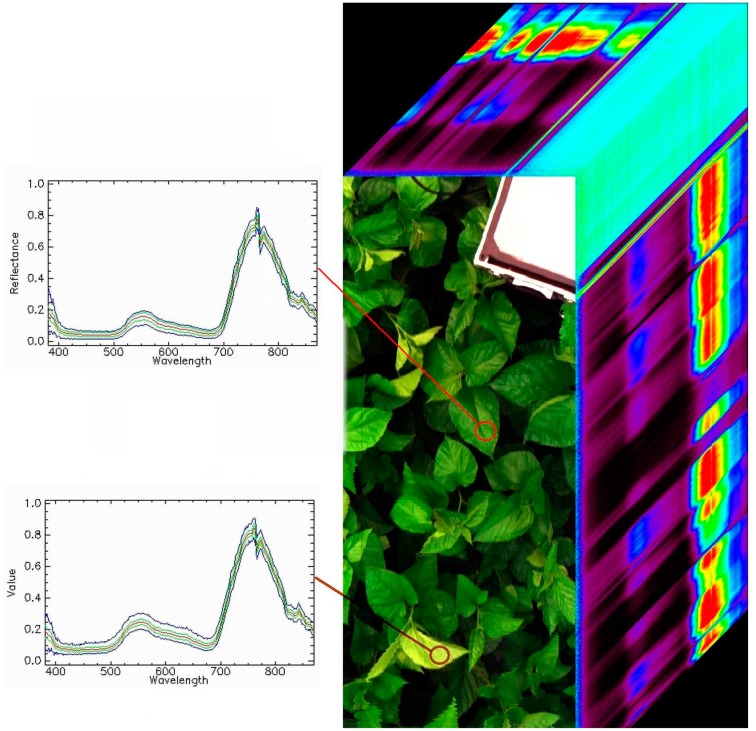
A sample of the hyperspectral data cube obtained by FISS.

The FISS acquires spatial data by rotating the scanning mirror, and the spatial resolution varies with the platform height. The optimal resolution is greater than 2 mm. Figure 3 shows the whole system, and the main technical specifications are listed in Table 1. Figure 4 shows a sample of the hyperspectral data cube obtained by FISS in a mulberry field [31,32].

### 2.2. Experimental Design

Sample collection: The experiment was conducted in the Huanjiang Observation and Research Station for Karst Ecosystem, Chinese Academy of Sciences. Several different fertilization gradients were set up, and fertilizers with different nutrient gradients were applied in an experimental soybean plot with the aim of studying nutrients in rain-fed lands. A total of 101 soybean plots were chosen to cover all nutrient gradients. Both new and old leaves were picked from the plants on 20 August 2009, and then stored in a box with ice and transported back to the laboratory within two hours.

Data acquisition: The collected leaves were removed from the cooler and laid flat on the laboratory bench. Soybean leaves were placed to make the surface as flat as possible. The imaging spectral data of the soybean leaves were then obtained with the FISS and an artificial cold light source (halogen light). The leaf was quickly returned to the cooler after measurement and taken to the chemical analysis laboratory for processing.

Chlorophyll extraction: The chlorophyll content was determined using the rapid extraction method with an ethanol-acetone mixture (1:1 by volume). Fresh leaves were cut into pieces, and 0.2 g of the pieces were weighed and placed in a 25-mL colorimetric tube for extraction for 18–24 h in the dark. The tube was shaken once every hour; the absorbance was measured with a UV spectrophotometer and used to calculate the contents of chlorophyll a, chlorophyll b and carotenoids.

**Figure 5.**
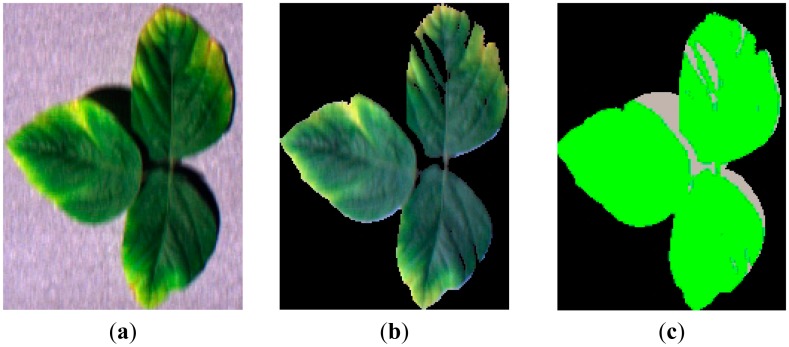
Shadow removal of soybean leaf data. (**a**) Raw data; (**b**) separated leaf; (**c**) shadow mask.

Data preprocessing: The reflectance images were obtained through a comparison with a gray standard panel. Namely, spectral reflectance was calculated as the ratio of leaf spectrum to the standard panel spectrum. The leaves were then separated from the background according to the threshold set, and shadows on the leaves were separated and removed (Figure 5). Because the lighting condition was stable in the lab, the contrast of spectra between leaf and shadow was quite remarkable. Leaves had much higher reflectance values of green wavelengths, while shadow had much lower reflectance values. Here, we use the reflectance value of the green band (550 nm) as the indicator and set a threshold to separate the leaf and shadow. This processing was performed using the software, ENVI 4.2. The valid extracted image data were then used for calculation and analysis.

## 3. Methods

### 3.1. General Description

The aim of this study is to evaluate the performance of the FISS through estimation of plant leaf chlorophyll contents. Whether this new system can be used for quantifying chlorophyll or even outperform traditional non-imaging spectrometer becomes the focus. As a typical non-imaging spectrometer, ASD is widely used in the remote sensing community to perform spectral experiments and quantify biochemicals, including chlorophyll. It can serve as a good counterpart to the FISS in spectral quantitative analysis. Detailed comparative analyses of chlorophyll estimation based on the two different spectrometers are presented in Section 4. Derivative spectra and reflectance spectra are used to estimate chlorophyll contents with ASD and FISS data. The same analysis methods and techniques are applied on both FISS and ASD data to make the comparison sound and comprehensive.

### 3.2. Model Features Used for Chlorophyll Content Estimation

In this section, the model feature is the information that can be used to retrieve the nitrogen content of the pigment and primarily includes the reflectivity of the raw spectra and derivative spectra.

The derivative spectra can enhance the subtle changes in the slope of the spectra; for vegetation, such changes are related to the biochemical absorption characteristics. The ability to enhance these changes can also help eliminate certain interfering factors and can better reflect the essential characteristics of vegetation. Therefore, derivative spectral images are calculated in the present study to obtain the derivative of the leaf spectra as follows:

(1) $$DR_{k}=R_{k}-R_{k-1}$$

where *DR_k_* is the first spectral derivative of the *k*-th wavelength, *R_k_* is the reflectance of the *k*-th wavelength and k is the serial number of the wavelength.

Because the spectral range of FISS is 380–870 nm, the same range of spectral data of ASD is selected and used in the study to maintain the comparability of the two sensors.

### 3.3. Spectral Analysis Models for Chlorophyll Content Estimation

To retrieve the chlorophyll content and compare different estimation methods, we used linear regression (LR), stepwise multiple linear regression (SMLR), partial least squares regression (PLSR) and support-vector machine (SVM) regression, the latter of which has become popular in recent years for processing high-dimensional small-size datasets. The following is a brief introduction to these methods.

(1) LR: LR is the simplest regression method. It assumes that the independent and dependent variables are linearly related, and the coefficient of the regression equation is estimated using the least squares method. This method is suitable for univariate analysis. Although it is simple, LR is the most commonly used estimation model.
(2) SMLR: Spectral analysis often involves a large number of highly-correlated wavelengths, and the high correlation between the variables usually results in unstable MLR models. SMLR is widely used for regression analysis and is a common method to reduce the correlation between independent variables. This method draws on the strengths of forward selection and backward elimination, while overcoming their drawbacks, and it also has a low computation load. SMLR can guarantee the production of an “optimal” regression equation for a specific significance level. The main parameters are the confidence level of the variable introduced (*α_in_*) and the confidence level of the variable deleted (*α_out_*). SMLR is implemented in SPSS13.0 [33].
(3) PLSR: PLSR can be used for regression modeling (multiple linear regression), to simplify the data structure (principal component analysis) and to analyze the correlation between two variables (canonical correlation analysis) [33]. This method proposes a breakthrough in multivariate statistical analyses. PLSR is a regression modeling method for multiple dependent variables *versus* multiple independent variables, and thus, it can solve numerous problems that conventional multiple regression is not able to solve. For example, PLSR can be used for solving problems involving multiple variables with a small sample size. PLSR is a nonlinear iterative method and usually outperforms the multiple linear regression method in processing data with mutual interferences between non-linear systems and quality parameters. In the present study, PLSR is implemented using the software, The Unscrambler 9.8.
(4) SVM regression: SVM regression (SVR) was first proposed by Vapnik and is a classification algorithm for small-sized samples with minimal separation [34,35]. SVM uses kernel methods to solve the classification of nonlinear problems. The core idea is to project nonlinear problems from a low-dimensional feature space to a high-dimensional feature space, so that the projected data are linearly separable, which simplifies the problem of solving linear SVM classification problems. SVM can be considered an expansion of SVM classification: the value range of the categories in classification problems is expanded to continuous values. For a specific N-element data set, {(**x**_1_,*y*_1_), …(**x***_n_*,*y_n_*)} (*y_i_* ∊ *R*, **x_i_** ∊ *R^d^* (*i* = 1,2,…*N*), where **x***_i_* is an input variable and *y_i_* is the target output variable, the final SVM regression equation is as follows [34,35]:
  (2) $$f(x)=\sum_{i=1}^{n}(-\alpha_{i}+\alpha_{i}^{*})K(x_{i},x)+b$$
  where *K* is the kernel function, *α_i_* ≥ 0 is the Lagrange multiplier and *b* ∊ *R^d^* is the offset vector. A detailed introduction to the SVM method can be found in the literature [34,35]. The ability of SVM to process high-dimensional data fits well with the high dimensional nature of imaging spectral data, and thus, it has a wide range of applications in processing imaging spectral data. In the present study, SVM is implemented in the Python language using the LIBSVM package [36].

The performance of the above 4 models can be measured with the coefficient of determination (COD) and root mean square error (RMSE), which are defined as follows:

(3) $$R^{2}=\sum_{i=1}^{n}{\left({\hat{y}}_{i}-\bar{y}\right)}^{2}/\sum_{i=1}^{n}{\left(y_{i}-\bar{y}\right)}^{2}$$

(4) $$RMSE=\sqrt{\sum_{i=1}^{n}{\left({\hat{y}}_{i}-y_{i}\right)}^{2}/n}$$

where *n* is the number of samples, *ȳ* is the measured mean of the sample and *ŷ* is the predicted value.

## 4. Results and Discussion

### 4.1. Spectral Variables as an Indicator of Pigment Content

**Figure 6.**
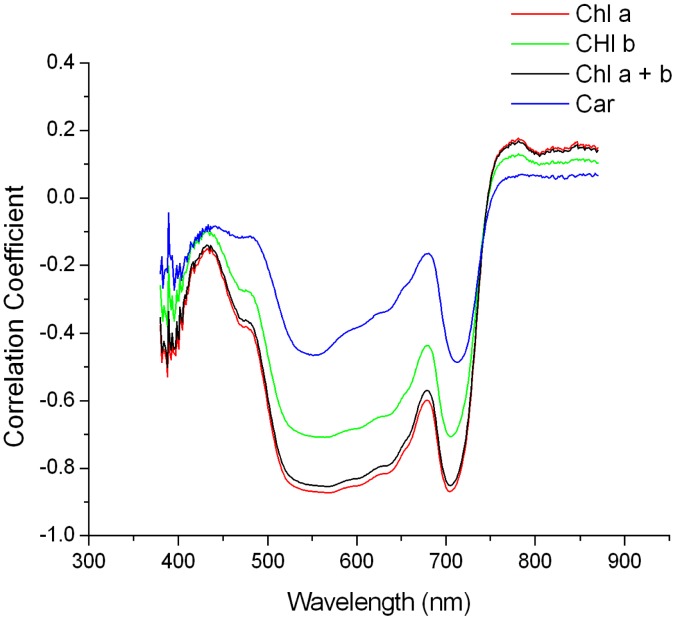
Correlogram of the mean spectral reflectance *versus* pigment content.

**Figure 7.**
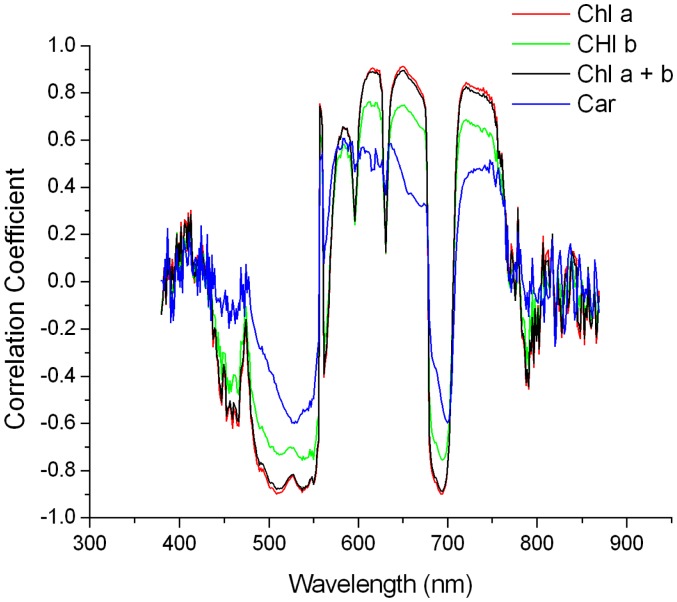
Correlogram of the mean derivative spectral reflectance *versus* pigment content.

To evaluate the utility of various spectral features as pigment content indicators, we calculated the linear correlation coefficients between these features and the pigment content (shown in Figures 6 and 7).

In both figures, the correlation coefficient curves of chlorophyll a, chlorophyll b, total chlorophyll and carotenoids were similar in shape, but had different ranges, and they coincided in wavelength with the high correlation coefficients, which will be illustrated in the following section using total chlorophyll as an example.

For the spectral reflectance, the wavelength ranges that exhibited high correlation coefficients were 530–670 nm and 695–715 nm, and the maximal correlation with chlorophyll content was −0.85 (corresponding to wavelengths of 568 nm and 703 nm). For the derivative spectra, there were four regions with relatively high correlation coefficients: 490–550, 600–670, 680–710 and 725–775 nm. As shown in Figures 6 and 7, the maximal correlation coefficient between the mean derivative spectral reflectance of the FISS data and chlorophyll content was −0.90 (corresponding to a wavelength of 650 nm). A comparison of the two figures revealed that the correlation coefficient between the FISS spectral derivative and pigment content was overall higher than that between the spectral reflectance and pigment content, indicating that the derivative spectral information was a better pigment content indicator.

### 4.2. Chlorophyll Content Retrieval

There were 101 valid samples for chlorophyll retrieval, which included 71 training samples and 30 validation samples. The maximum, minimum, mean and standard deviation of the chlorophyll content of the training samples were 5.33, 2.33, 4.19 and 0.75 mg/g, respectively, and the corresponding values for the validation samples were 5.41, 2.69, 4.05 and 0.76 mg/g, respectively. The statistics of the training samples and validation samples were generally the same; therefore, these samples can be used for the construction and validation of the same model.

For different spectral features (spectral reflectance, derivative spectra), significant wavelengths were selected using stepwise methods, and then, the chlorophyll content was retrieved using the LR, MLR, PLSR and SVM models. The retrieval accuracies are shown in Table 2 (FISS data). The LR model used the first significant wavelength; the MLR model used the first three and first seven wavelengths; the PLSR model used all of the wavelengths; and the SVM model used the same wavelengths as those in the MLR model, as well as all of the wavelengths. The number of features and accuracy in each case are shown in Table 2 for comparison.

ASD spectrometer was used as well to obtain the spectra data of these samples simultaneously. Similar methods mentioned above were also performed on ASD data sets. Features and accuracy in each case based on ASD data are shown in Table 3.

#### 4.2.1. Comparative Analysis of the Accuracy of Chlorophyll Content Retrieval Based on Imaging Spectrometer and Non-Imaging Spectrometer

From the comparison of Table 2 with Table 3, it is easy to find that chlorophyll content retrieval accuracies based on the imaging spectrometer are higher than those based on a traditional single-point sensor spectrometer. In the case where spectral reflectance is selected as the model features, the lowest RMSE is 0.348 mg/g based on ASD data, while the lowest RMSE is 0.222 mg/g based on FISS data. The retrieval error generates a relative reduction of 36.2%. A similar result can be found in the case where derivative spectral reflectance is the model feature. For example, the MLR retrieval error of the validation samples at seven wavelengths is 0.201 mg/g based on FISS data, a relative reduction of 46.8% compared with the error 0.378 mg/g based on ASD data.

The imaging spectrometer could obtain both spectral and spatial detailed information. Its image-spectrum-in-one merit can provide more information about the whole leaf and is more conducive to chlorophyll content estimation. Meanwhile, the imaging spectrometer can accurately obtain the spectral information of any valid pixel in the entire leaf and accurately locate the target. A single-point sensor spectrometer can only obtain information in the sensor's field of view and not the complete spectral information of the entire leaf. This could lead to the fact that its spectral signals cannot be precisely mapped to the target objects. In our cases, the entire leaf was used for chlorophyll extraction, while we cannot get the complete spectral information of the entire leaf using a non-imaging spectrometer. The imaging spectrometer can overcome this shortcoming and, thus, obtain better results for chlorophyll content estimation.

#### 4.2.2. Comparative Analysis of the Accuracy of Chlorophyll Content Retrieval Based on Different Spectral Features

The comparison between the retrieval accuracies of the spectral reflectance and derivative spectra of the FISS data indicated that when the same method and same number of variables are used (except for the PLSR model, which used all of the wavelengths), the derivative spectra provided a higher accuracy of chlorophyll content estimation: its maximal validation accuracy in RMSE was 0.201 mg/g, and its coefficient of determination was 0.94. The highest prediction accuracy of the spectral reflectance in RMSE was 0.222 mg/g, and its coefficient of determination was 0.92. Using the derivative spectrum can reduce the RMSE by 3.3%–35.6%. When only a small number of wavelengths were used, the difference was particularly significant, and the mean reduction was 22.7%. This result demonstrates that the derivative spectrum is a more sensitive chlorophyll content indicator that can extract content information more efficiently than the raw spectral reflectance.

From Table 3, it can be found that the derivative spectrum serves as a better indicator of chlorophyll content generally compared to raw spectral reflectance for ASD data. It holds true for both FISS and ASD data that derivative spectra have better performance than reflectance spectra for quantifying chlorophyll. However, this phenomenon is much more significant for the FISS data. Significant reductions of the estimation RMSEs were observed by comparing the results derived from derivative and reflectance spectra of the FISS data. However, the corresponding improvements are not so obvious for ASD data by the fact that RSMEs reductions are mainly observed in the calibration dataset, while no significant reductions are found in the validation dataset in our cases.

If all 344 of the wavelengths are used in the models, then the advantages of using the derivative spectra become less significant, because the derivative spectra are essentially a method of enhancing information, while effectively suppressing noise at specific wavelengths. Therefore, the derivative spectra do not increase the amount of information carried by the FISS data. When all of the wavelengths are used, the spectral reflectance and derivative spectra carry the same amount of information. Consequently, the derivative spectra cannot significantly improve the estimation accuracy with all bands used. This point holds true as well for ASD data due to similar reasons.

#### 4.2.3. Comparative Analysis of the Accuracy of Chlorophyll Content Retrieval Using Different Regression Models

For spectral reflectance, the highest prediction accuracy was achieved when using the information at all of the wavelengths in the PLSR model, which had a corresponding RMSE of 0.222 mg/g. For the derivative spectra, the smallest prediction error was 0.201 mg/g, which occurred when using the information of seven wavelengths in the MLR model (Table 2). Among the four models, the accuracy achieved by the LR model was generally the lowest because the LR model used fewer characteristic variables and, therefore, had insufficient information.

When using three or seven wavelengths, the SVM (a nonlinear method) did not outperform the MLR model, indicating that the relationship between the chlorophyll content and these significant wavelengths was closer to a linear relationship than to a complex non-linear relationship. Thus, nonlinear models did not display an advantage and might present the risk of over-learning.

When using the information at all 344 wavelengths, the performance of the PLSR was improved, and the RMSE was 9.8% lower than that of the SVM model. However, when using the derivative spectra at all of the wavelengths, the estimation errors of the SVM and PLSR models were almost the same, which may imply that using sophisticated methods does not always increase the estimation accuracy and that selecting the appropriate spectral feature (e.g., spectral reflectance or derivative spectra) may be more important.

Therefore, we can conclude that regression methods only require information at several significant wavelengths to retrieve chlorophyll content, and they can be used in multivariate linear models. Specifically, this conclusion holds true as well for ASD data by comparing the different regression models (Table 3).

## 5. Conclusions

An FISS was used to study the retrieval of soybean leaf chlorophyll content in the present study, and the performance of the FISS system in a quantitative spectral analysis was assessed. The main conclusions are as follows.

(1) For the raw spectra, reflectance in the ranges of 530–670 and 695–715 nm exhibited a strong correlation with pigment content; for the derivative spectra, the following four regions showed relatively high correlation coefficients: 490–550, 600–670, 680–710 and 725–775 nm.
(2) Compared with the spectral reflectance, the derivative spectra displayed a stronger correlation with pigment content and were a better indicator of pigment content, which is more significant for FISS data compared to ASD data. Using derivative spectra as the input feature in regression models resulted in a much higher accuracy than when the raw spectral features were used, with the RMSE reduced by 3.3%–35.6%. This difference was more significant when only a small number of wavelengths were used, with a mean reduction of 22.7%.
(3) Different modeling methods presented different accuracies, although the overall differences were not significant. The impact of the model selection on accuracy was smaller than that of the spectral feature. Regression methods only required information from several significant wavelengths to retrieve chlorophyll content, and they can be used in multivariate linear models. This conclusion holds true for both FISS and ASD data.
(4) Because of its unique measurement method and image-spectrum-in-one feature, FISS data can be used to acquire accurate chlorophyll content information with higher accuracies than non-imaging spectral data. The relative reduction of retrieval RMSE could reach a level of more than 30%. The lowest retrieval RMSE based on FISS data was 0.201 mg/g, which indicates a high estimation accuracy compared with the mean chlorophyll content of the sampled leaves (4.05 mg/g), confirming the excellent performance of FISS in quantitative spectral analyses. Therefore, FISS can be widely used in the agricultural sector.

## Figures and Tables

**Table 1.** Main parameters and specifications of the FISS.

**Main Parameters**	**Values**
Band number	344
Spectral range	379–870 nm
Spectral resolution	4–7 nm
Spatial resolution	≥2 mm
Radiance calibration error in laboratory	≤5%
Imaging rate/(lines/s)	20
Scan field/°	−20–20
Quantitative value /bit	12
Signal to noise	>500 (60% of bands)
Spectral sampling interval/nm	About 1.4

**Table 2.** Spectral variables and chlorophyll content retrieval accuracies for the different models (FISS data) (RMSEc refers to RMSE of calibration dataset and RMSEv refers to RMSE of validation dataset).

**Feature**	**Model**	**No. of Wavelengths**	**Calibration**		**Validation**	
	
			**RMSEc**	*R*^2^	**RMSEv**	*R*^2^
spectral reflectance	LR	1	0.404	0.71	**0.310**	0.85
	MLR	3	0.302	0.84	0.297	0.86
	MLR	7	0.259	0.88	0.312	0.85
	PLSR	344	0.244	0.90	**0.222**	**0.92**
	SVR	3	0.282	0.86	0.352	0.82
	SVR	7	0.277	0.86	0.276	0.89
	SVR	344	0.262	0.88	0.246	0.91

derivative spectral reflectance	LR	1	0.339	0.79	**0.254**	0.90
	MLR	3	0.283	0.86	0.254	0.90
	MLR	7	0.258	0.88	**0.201**	**0.94**
	PLSR	343	0.275	0.86	0.236	0.90
	SVR	3	0.262	0.88	0.266	0.90
	SVR	7	0.270	0.87	0.241	0.92
	SVR	343	0.239	0.90	0.238	0.91

**Table 3.** Spectral variables and chlorophyll content retrieval accuracies for the different models (ASD data).

**Feature**	**Model**	**No. of Wavelengths**	**Calibration**		**Validation**	
	
			**RMSEc**	*R*^2^	**RMSEc**	*R*^2^
spectral reflectance	LR	1	0.665	0.20	0.527	0.57
	MLR	3	0.510	0.53	0.380	0.77
	MLR	7	0.408	0.70	**0.348**	0.80
	PLSR	520	0.364	0.76	0.364	0.78
	SVR	3	0.464	0.61	0.405	0.74
	SVR	7	0.403	0.71	0.405	0.72
	SVR	520	0.399	0.74	0.412	0.71

derivative spectral reflectance	LR	1	0.581	0.39	0.424	0.72
	MLR	3	0.477	0.59	0.413	0.76
	MLR	7	0.365	0.76	**0.378**	0.78
	PLSR	520	0.489	0.57	0.406	0.72
	SVR	3	0.344	0.79	0.463	0.73
	SVR	7	0.380	0.75	0.411	0.77
	SVR	520	0.381	0.76	0.431	0.71
